# Stroke risk and its association with quality of life: a cross-sectional study among Chinese urban adults

**DOI:** 10.1186/s12955-021-01868-9

**Published:** 2021-10-09

**Authors:** Huiqing Yao, Juhua Zhang, Yanmei Wang, Qingqing Wang, Fei Zhao, Peng Zhang

**Affiliations:** 1 Clinical Trial Center, Beijing Hospital, National Center of Gerontology, Institute of Geriatric Medicine, Chinese Academy of Medical Science, Beijing Key Laboratory of Drug Clinical Risk and Personalized Medication Evaluation, Beijing, 100730 People’s Republic of China; 2 Fudan University, Shanghai, 200433 People’s Republic of China; 3 Shanghai Pudong Health Development Research Institute, Shanghai, 200129 People’s Republic of China; 4 Shanghai University of Medicine and Health Sciences Affiliated Zhoupu Hospital, Shanghai, 201318 People’s Republic of China; 5 Department of Nursing, Shanghai Gongli Hospital, Second Military Medical University, Shanghai, 200135 People’s Republic of China; 6 Department of Neurology, Jiading District Central Hospital Affiliated Shanghai University of Medicine and Health Sciences, Shanghai, 201800 People’s Republic of China; 7 School of Clinical Medicine, Shanghai University of Medicine & Health Sciences, Shanghai, 201318 People’s Republic of China

**Keywords:** Stroke, Risk, Quality of life, Physical inactivity, Hypertension, Smoking, China

## Abstract

**Background:**

Stroke is a leading cause of mortality and disability worldwide. Most stroke risk studies focused on more established biological and pathophysiological risk factors such as hypertension and smoking, psychosocial factors such as quality of life are often under-investigated and thus less reported. The current study aims to estimate stroke risk and explore the impact of quality of life on stroke risk among a community sample of urban residents in Shanghai.

**Methods:**

This cross-sectional study was conducted in Fengxian District of Shanghai City from December 2018 to April 2019. 4030 representative participants were recruited through a multistage, stratified, probability proportional to size sampling method and completed the study. Stroke risk was assessed using the Rapid Stroke Risk Screening Chart that included 8 risk factors for stroke. Quality of life was measured using the World Health Organization Quality of Life-brief version (WHOQOL-BREF).

**Results:**

One-third of residents were at risk for stroke, including 14.39% at high risk, and 18.68% at middle risk. The top three most commonly reported risk factors were physical inactivity (37.30%), hypertension (25.38%), and smoking (17.32%). Quality of life and its four domains were all independently and significantly associated with stroke risk. Multinominal logistic regressions showed that a one-unit increase in the quality of life was associated with a decreased relative risk for middle-risk relative to low-risk of stroke by a factor of 0.988 (95% CI:0.979, 0.997, *P* = 0.007), and a decreased relative risk for high-risk relative to low-risk of stroke by a factor of 0.975 (95% CI:0.966, 0.984, *P* < 0.001).

**Conclusions:**

Our findings showed an alarmingly high prevalence of stroke risk among the sample, which may require future intervention programs to focus on improving both biological and behavioral risk factors such as increasing physical activity, early diagnosis and treatment of hypertension, and smoking cessation, as well as improving psychosocial factors such as quality of life.

## Background

Stroke has emerged as a major global public health challenge and is a leading cause of mortality and disability throughout the world [1, 2]. According to the 2016 Global Burden of Diseases, Injuries, and Risk Factors Study (GBD) [3], stroke constituted the second largest cause of global death with 5·5 million deaths, and also the second most common cause of global disability-adjusted life-years (DALYs) with 116·4 million DALYs. In 2016, there were 80.1 million prevalent cases of stroke worldwide, with 13.7 million new stroke cases occurring in the same year [3]. Although the global age-standardized mortality rate and DALY rate has decreased from 1990 to 2016, the absolute cases of stroke have increased sharply all over the world. The global burden of stroke remains high, causing substantial economic and social costs for stroke treatment and post-stroke care [3, 4]. Such a situation was further worsened by the global aging population, transition from infectious to non-communicable diseases of most low and middle-income countries, as well as globally increasing modifiable risk factors for stroke [5]. It is expected that stroke burden will increase in the next few decades, especially in developing countries like China.

China has the highest age-standardized incidence of stroke in the world, with 354 new cases per 100 000 person-years [3]. In 2016, stroke has led to a direct cost of hospitalization of 85.5 billion Renminbi (equivalent to 12.2 billion in US dollar) [6]. Stroke has become a leading cause of death and disability in China [3, 4]. The high prevalence and incidence of stroke in China not only causes great suffering to the affected person, but also brings about substantial burden to their family and the society. On the personal level, stroke brings about serious negative consequences in physical, mental and social aspects of life, leading to disability in almost 90% of those afflicted [7]. On the family level, stroke causes huge caregiver burden to family members, who are usually unprepared to accept the sudden disabilities of their loved ones and deal with their long process of rehabilitation [8]. On the society level, both the direct financial cost from stroke treatment and rehabilitation care, as well as the indirect cost of lost productivity from both the affected and their caregivers are tremendous, leading to high social burden [6].

Considering the high prevalence and huge disease burden of stroke, it is both urgent and crucial to identify and manage risk factors for stroke to guide for timely and early stroke prevention, which has positive impacts on the individual, family and the society [9]. A large number of risk factors of stoke has been identified by the past literature, which may be generally classified into modifiable and non-modified factors based on whether they are subject to change or intervention [9–14]. Non-modifiable risk factors may include age, gender, ethnicity, genetic attributes, and family history. Modifiable risk factors may include physiological components such as body mass index (BMI), adiposity, hypertension, dyslipidemia, diabetes mellitus, atrial fibrillation (AF), as well as lifestyle behaviors such as smoking, physical inactivity, poor diet, and high alcohol consumption [9–14].

The association between psychosocial factors such as quality of life and stroke risk has also been widely studied, with most studies showing that people’s quality of life significantly decreased after stroke [8, 15–17]. Although quality of life has been frequently assessed as a poststroke outcome, evidence has shown that quality of life also serves as an important predictor for some major diseases such as myocardial infarction (MI), heart failure, diabetes mellitus, and hemodialysis [18–23]. Using a case–control study design, Egido et al. [24] found that psychosocial stress predicted stroke, indicating a causal link between quality of life and stroke, which was further supported by Shams et al.’s [25] study. Using a large-scale prospective study design, Shams et al. [25] found that lower baseline quality of life was a strong predictor for later risk of stroke, with a 20% increased stroke risk in the lowest baseline quality of life quartile as compared to the highest quartile. Evidence from past studies suggest bidirectional relationships between stroke and quality of life, and quality of life may serve as both a predictor and an outcome of stroke. As an important modifiable psychosocial factor, quality of life warrants research attention in stoke studies, which may provide significant clinical and political implications in guiding for future programs in stroke risk prevention and after-care.

The current study was conducted with two major aims: (1) To examine stroke risk among a representative community sample of adult residents in urban China, such as the proportion of middle and high risk of stroke, and the leading risk factors of stroke; (2) To explore the association between quality of life and stroke risk. We hypothesize that stroke risk is high among Chinese urban community residents, and that there is significant negative association between quality of life and stroke risk, with lower quality of life associated with higher risk of stroke.

## Methods

### Participants

**Fig. 1:**
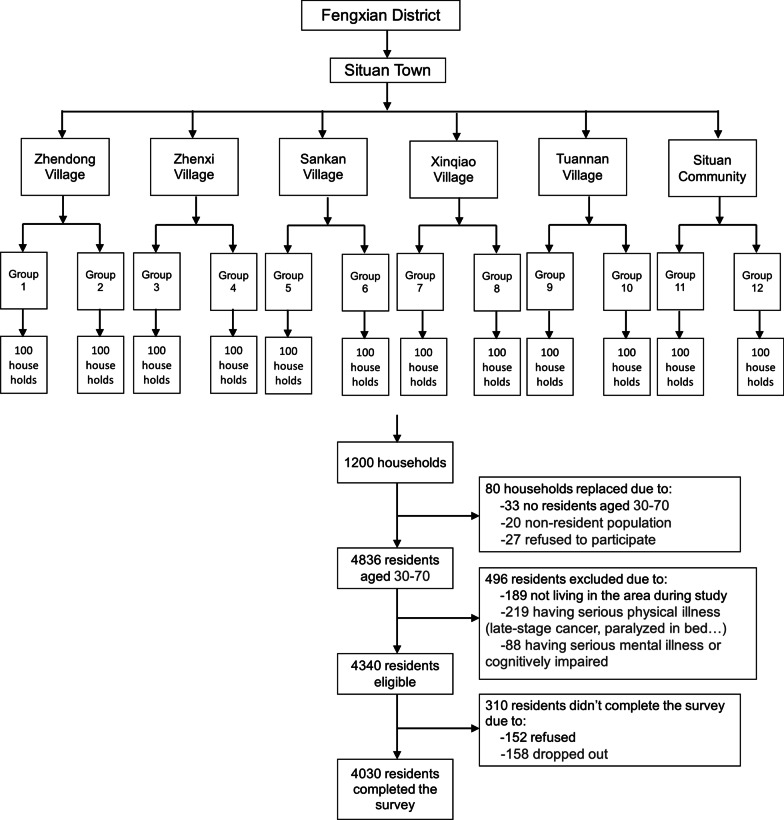
Flowchart of subject enrollment

The cross-sectional study was conducted in Fengxian District of Shanghai Urban areas. The target population was residents aged 30–70 years who had lived in the urban areas of Fengxian District, Shanghai City, for over 6 months. The survey was conducted from December 2018 to April 2019.A multistage, stratified, probability proportional to size sampling was adopted to identify participants. In the first stage, one town was randomly selected from eight towns of Fengxian District. In the second stage, six neighborhood committees/ administrative villages were randomly selected from the town. In the third stage, two resident/villager groups that include more than 100 households were randomly selected from each neighborhood committee/ administrative village. In the fourth stage, 100 households were randomly selected from each resident/villager group. In the field investigation, if the selected households do not meet the inclusion criteria (such as no residents over 30 years old, non-resident population) or are unwilling to accept the investigation, the households need to be replaced. In the process of replacement, residents who were not selected from the same resident /villager group as the investigation households or from adjacent resident/villager groups were selected according to the principle of living nearby. The family structure of the replacement households should be similar to that of the original households. The percentage of replacement should not exceed 20%. Based on this criteria, 80 households out of the total 1200 households were replaced, leading to a replacement rate of 6.67%. In this stage, 4836 residents aged 30–70 were selected from the 1200 households. Those who were not living in the areas during the research period, those with difficulty in communication due to serious physical or mental illness and those who were cognitively impaired or actively psychotic, were excluded. Based on the exclusion criteria, 496 residents were excluded, leading to 4340 eligible residents. Among the 4340 residents approached for study participation, 310 didn’t complete the study due to refusal of participation or dropout during the study, leading to a final sample of 4030 residents with a response rate of 92.86%. Figure 1 shows the flow chart of participant enrollment.

### Procedure

The study was approved by the Ethics Review Committee of the Shanghai University of Medicine & Health Sciences. Interviewers were medical students in the Shanghai University of Medicine & Health Sciences, and they all received a one-week uniform formal training to conduct the interviews prior to the formal study. Interviewers were accompanied by the Village cadres to visit each household and explained in detail the purpose and process of the study to each eligible participant. Each participant was invited to answer a series of questionnaires after providing written informed consent. All questionnaires were filled in by the respondents themselves. The answers were then checked by a quality control person to ensure integrity, accuracy and consistency.

### Measures

*Risk of stroke* Risk of stoke was assessed using the Rapid Stroke Risk Screening Chart which collected 8 risk factors for stroke: hypertension, diabetes mellitus, dyslipidemia, atrial fibrillation, current smoking, overweight or obesity, physical inactivity, and family history of stroke. According to the China National Stroke Screening Survey (CNSSS) [26], the definition of each risk factor was as follows: Hypertension refers to a self-reported history of hypertension or the use of antihypertensive drugs, or the average of two resting systolic blood pressure readings of ≥ 140 mmHg and/or diastolic blood pressure ≥ 90 mmHg in the field survey [27]. Diabetes mellitus refers to the use of insulin and/or oral hypoglycaemic medications, or a self-reported history of diabetes or FBG ≥ 7.0 mmol/L in the field survey [28]. Dyslipidemia refers to the use of a lipid-lowering medication or having at least one of the following in the field survey: triglycerides (TG) ≥ 1.70 mmol/L, cholesterol (TC) ≥ 5.18 mmol/L, and low-density lipoprotein cholesterol (LDL-C) ≥ 3.37 mmol/L [29]. Atrial fibrillation was defined as reported by the respondent or diagnosed by ECG in the field survey. Current smoking refers to smoking at least one cigarette per day by self-report. Overweight or obesity was defined by calculating body mass index (BMI) as weight (kg) divided by height squared (m^2^), with BMI ≥ 28 kg/m^2^ indicating overweight or obesity [30]. Physical inactivity was defined as less than three times a week of physical exercise that lasts less than 30 min including industrial and agricultural labour [31]. A family history of stroke was restricted to immediate family members. For each risk factor, the participants answered “yes” or “no”, with each “yes” answer scored 1 point, while each “no” answer scored 0 point. Participants with ≥ 3 above-mentioned risk factors were classified into high-risk group. Participants with < 3 above-mentioned risk factors were further classified into middle-risk and low-risk group based on whether they had either of the following three risk factors: hypertension, diabetes mellitus, atrial fibrillation. Participants with any one of the three above-mentioned risk factors were classified into middle-risk group. Participants with none of the three above-mentioned risk factors were classified into low-risk group.

*Quality of Life* Quality of life was assessed using the World Health Organization Quality of Life-brief version (WHOQOL-BREF), which is a short version of the original WHOQOL-100 developed by the WHO [32]. The WHOQOL-BREF is a self-report questionnaire to assess quality of life in various domains. As a broad and comprehensive tool, the WHOQOL-BREF is available in 19 different languages and has been proven to be cross-culturally applicable. It contains 26 questions with the first 2 questions asking about the overall quality of life and overall perception of their own health status. The next 24 questions ask about individuals’ perceived health in 4 domains: physical health (7 items), psychological health (6 items), social relationships (3 items), and environment (8 items). Each item is rated on a 5-point scale ranging from 1 = “low, negative perception” to 5 = “high, positive perception”. The domain score was calculated by mean score of items within each domain, which were then multiplied by 4 to keep comparable with the scores used in the WHOQOL-100. The Chinese version of WHOQOL-BREF used in the present study demonstrated good internal consistency, with a Cronbach's α coefficient of 0.95.

### Statistical analysis

All statistical analyses were conducted using SPSS version 24.0 [33]. Multiple imputations were used for missing values. Frequency and percentages were displayed for categorical variables, and mean, standard deviation, and range displayed for continuous variables. Oneway ANOVA test was used to compare socio-demographic and quality of life differences among three levels of risk of stroke, followed by post hoc analysis for significant statistics. Since the same sample was compared for 9 times, we used Bonferroni corrections by dividing 0.05 by 9 and set alpha value as 0.0056 to adjust for Type I error. Several multinominal logistic regression analyses were performed with stroke risk as dependent variable, and quality of life and its four domains as independent variable separately, while controlling for socio-demographics. Odds ratio and 95% confidence intervals (CI) were reported, with *P* values of 0.05 considered statistically significant.

## Results

### Sample characteristics

A total of 4030 participants completed the questionnaire. The Participants had an average age of 52.22 ± 11.21, ranging from 30 to 70. There were more were females than males (54.81% vs 45.19%). Most were married (93.50%), and the largest proportion of participants had an education level of middle and high school (49.26%), followed by college and above (28.31%).

### Stroke risk

Table 1 shows the prevalence of each of the eight risk factors in descending order. The most frequently reported risk factor was physical inactivity (37.30%), followed by hypertension (25.38%) and smoking (17.32%). The least commonly reported risk factor was atrial fibrillation, with only 52 participants reporting atrial fibrillation (1.29%). Combining the eight risk factors, participants were further classified into three risk groups, with most falling into the category of low-risk group (66.92%). However, there were still 14.39% in the high-risk group, and 18.68% in the middle-risk group of stroke, leading to almost one third (33.07%) of the population at risk for stroke.

**Table 1:** Eight risk factors for stroke

Characteristics		n (%)
*Risk factors for stroke*		
Physical inactivity	No	2527 (62.70)
	Yes	1503 (37.30)
Hypertension	No	3007 (74.62)
	Yes	1023 (25.38)
Smoking	No	3332 (82.68)
	Yes	698 (17.32)
Dyslipidemia	No	3602 (89.38)
	Yes	428 (10.62)
Family history of stroke	No	3656 (90.72)
	Yes	374 (9.28)
Diabetes mellitus	No	3686 (91.46)
	Yes	344 (8.54)
Overweight or obesity	No	3752 (93.10)
	Yes	278 (6.90)
Atrial fibrillation	No	3978 (98.71)
	Yes	52 (1.29)
*Stroke risk level*	Low risk	2697 (66.92)
	Middle risk	753 (18.68)
	High risk	580 (14.39)

### Comparison among three stroke risk groups

**Fig. 2:**
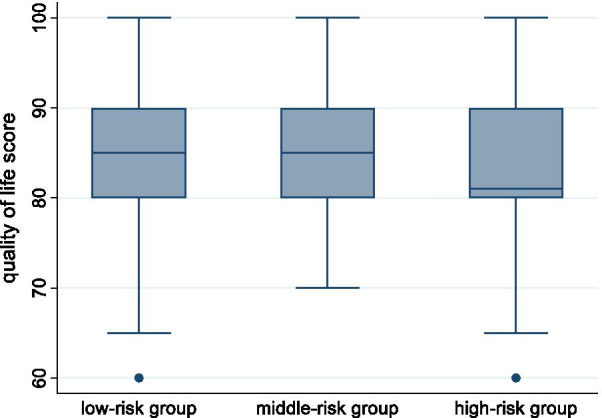
Boxplots of the quality of life scores among the three stroke risk groups

Table 2 shows the comparisons of socio-demographics and quality of life among low-risk, middle-risk, and high-risk groups for stroke. No significant difference was found in socio-demographic characteristics among the three groups. For quality of life, all three groups showed significant differences in both the total WHOQOL score (also shown in Fig. 2) and its four domains. The total quality of life was highest in the low-risk group, and lowest in the high-risk group, with significant difference between each two groups. For the four domains of quality of life, the low-risk group showed significantly higher scores than the high-risk group in physical health, psychological health, social relationships, and environment.

**Table 2:** Comparisons among three risk groups for stroke

Socio demographics	Low-risk (G1, n = 2697)	Middle-risk (G2, n = 753)	High-risk (G3, n = 580)	χ^2^/F	P*	Post-hoc analysis
Age	52.31 ± 11.23	51.97 ± 11.19	52.13 ± 11.19	0.30	0.738	
*Gender*						
Male	1222 (45.31)	342 (45.42)	257 (44.31)	0.21	0.899	
Female	1475 (54.69)	411 (54.58)	323 (55.69)	0.021	0.989	
*Education*						
Primary and below	603 (22.36)	184 (24.44)	117 (20.17)	5.64	0.228	
Middle and high school	1340 (49.68)	346 (45.95)	299 (51.55)			
College and above	754 (27.96)	223(29.61)	164 (28.28)			
*Marriage*						
Married	2525 (93.62)	701 (93.09)	542 (93.45)	0.27	0.872	
Unmarried	172 (6.38)	52 (6.91)	38 (6.55)			
*Quality of Life*						
Physical health	13.64 ± 1.89	13.53 ± 1.83	13.32 ± 1.91	7.18	< 0.001	G1 > G3
Psychological health	13.86 ± 2.30	13.63 ± 2.17	13.42 ± 2.19	10.54	< 0.001	G1 > G2, G1 > G3
Social relationships	15.40 ± 2.76	15.06 ± 2.67	14.71 ± 2.73	17.20	< 0.001	G1 > G2, G1 > G3
Environment	14.85 ± 2.65	14.77 ± 2.58	14.33 ± 2.55	9.28	< 0.001	G1 > G3, G2 > G3
Total score	84.90 ± 9.05	83.90 ± 8.90	82.61 ± 9.64	16.33	< 0.001	G1 > G2 > G3

*Alpha value was set at 0.0056 after Bonferroni corrections

### Correlates of stroke risk

Table 3 shows the results of five different multinominal logistic regression analyses with stroke risk as the dependent variable, the total score of quality of life and its four domains as independent variables, respectively, while controlling for all socio-demographic characteristics. In general, stroke risk was independently significantly associated with quality of life and its four domains. For the total score of quality of life, a one-unit increase in the quality of life was associated with a decreased relative risk for middle-risk relative to low-risk of stroke by a factor of 0.988 (95% CI:0.979, 0.997, *P* = 0.007), and a decreased relative risk for high-risk relative to low-risk of stroke by a factor of 0.975 (95% CI:0.966, 0.984, *P* < 0.001). For the four domains of quality of life, increase of social health and mental health was associated with decreased relative risk of middle-risk vs low-risk and high-risk vs low risk, while increase of physical health and environment was only associated with decreased relative risk of high-risk vs low risk of stroke.

**Table 3:** Multinominal logistic regression of quality of life on stroke risk

	Correlates	Coefficients (95% CI)	Relative risk (95% CI)	z	P
Low-risk group	Reference	–	–		
Middle-risk group	Quality of life	− 0.012 (− 0.021, − 0.003)	0.988 (0.979, 0.997)	− 2.7	**0.007**
	Physical health	− 0.032 (− 0.075, 0.011)	0.968 (0.928, 1.010)	− 1.46	0.144
	Mental health	− 0.044 (− 0.080, − 0.008)	0.957 (0.923, 0.992)	− 2.42	**0.015**
	Social health	− 0.045 (− 0.075, − 0.016)	0.956 (0.928, 0.984)	− 3.01	**0.003**
	Environment	− 0.011 (− 0.042,0.019)	0.989 (0.959, 1.020)	− 0.73	0.466
High-risk group	Quality of life	− 0.025 (− 0.035, − 0.016)	0.975 (0.966, 0.984)	− 5.44	**< 0.001**
	Physical health	− 0.091 (− 0.14, − 0.43)	0.913 (0.870, 0.958)	− 3.7	**< 0.001**
	Mental health	− 0.087 (− 0.13, − 0.047)	0.917 (0.881, 0.954)	− 4.27	**< 0.001**
	Social health	− 0.093 (− 0.13, − 0.059)	0.912 (0.882, 0.942)	− 5.47	**< 0.001**
	Environment	− 0.076 (− 0.11, − 0.041)	0.927 (0.896, 0.960)	− 4.29	**< 0.001**

Adjusted socio-demographics included: age, gender, education, and marital status

## Discussion

### Summary of the findings

To our knowledge, this is the first community-based large population study to examine the risk of stroke and its association with quality of life among a representative sample of urban residents in China. The main findings of the study are that although over two thirds of residents (66.92%) were at low risk for stroke, there were still 14.39% at high risk for stroke, and 18.68% at middle risk. Among the eight risk factors for stroke, the top three were physical inactivity (37.30%), hypertension (25.38%), and smoking (17.32%). Multinominal logistic regressions showed that a one-unit increase in the quality of life was associated with a decreased relative risk for middle-risk relative to low-risk of stroke by a factor of 0.988 (95% CI:0.979, 0.997, *P* = 0.007), and a decreased relative risk for high-risk relative to low-risk of stroke by a factor of 0.975 (95% CI:0.966, 0.984, *P* < 0.001).

### Stroke risk

The finding that 14.39% participants were at high risk for stroke in the current study was slightly lower than the 17.1% reported by Yi et al. [10] in another large population-based study in southwestern China. This may be explained by the relatively younger age in the current study than Yi et al.’s [10] study, as risk of stroke has been consistently shown to increase with aging [34, 35]. However, when combining high-risk and middle-risk groups, over one third were at some risk of stroke, which is alarming and also consistent with the 2016 GBD study showing China has the highest age-standardized incidence of stroke in the world [3]. The high incidence and heavy disease burden of stroke indicates that it is both essential and urgent to control risk factors for stroke prevention.

Among the eight risk factors for stroke, physical inactivity, hypertension, and smoking were the top three common factors reported by the participants. The finding that 37.30% participants were physically inactive was consistent with Ng et al.’s [36] study showing Chinese Population in physical activity has decreased by 25% from 1991 to 2011. The high prevalence of physical inactivity is reflective of China's rapid urbanization and changes in working style and lifestyle. Numerous studies have showed physical inactivity as a strong predictor for stoke and stroke severity [37, 38]. One mechanism may be through modulation of endothelial function and vascular reactivity [39]. Considering physical activity is an easily modifiable factor, this finding has implications for development of various exercise programs to increase physical activity in community in order to reduce the risk of stroke.

In our study, 25.38% of participants had hypertension, a rate comparable to a recent study conducted in Guangdong of China [40]. Hypertension has been reported to be the most important contributor for stroke in the literature. The prevalence of hypertension has been increasing rapidly in recent years, yet the rates of awareness, treatment and control were still very low [41]. Our findings call for more public education on hypertension prevention and control in the community to decrease future risk of stroke.

Our study also showed a smoking rate of 17.32% among the participants, which is in keeping with Ng’s study showing significant decrease in the prevalence of smoking from 30.4% in 1980 to 24.2% in 2012 [42]. Smoking is a well-known risk factor for stroke, and also a modifiable factor that is subject to change. This finding has implications for future stroke prevention programs to focus on increasing public education and awareness on risk of smoking and developing smoking cessation programs in the community when necessary.

### Quality of life and stroke

Although abundant evidence has shown stroke led to lower quality of life [8, 15–17], few studies have explored the opposite direction–-the impact of quality of life on stroke. Using stroke risk as dependent variable, and quality of life and its four domains as dependent variables, our study has consistently shown that lower quality of life (including lower physical health, psychological health, social relationships, and environment) was associated with higher risk of stroke in multinominal logistic regressions. Our results lent further support to Shams et al.’s [25] finding that lower baseline quality of life predicted later risk of stroke. Compared to the more established biological and pathophysiological risk factors for stroke such as physical inactivity, hypertension and smoking, psychosocial factors such as quality of life are often under-investigated and thus less reported [43, 44]. However, psychosocial factors are equally important in contributing to stoke and other cardiovascular diseases and need more research attention.

So far, the underlying mechanisms connecting psychosocial factors to stroke risk are complex and not fully elucidated. Possible explanations involve vascular inflammation, oxidative stress or immune dysfunction caused by psychosocial risk factors that lead to pathophysiology of vascular disease [45]. Besides, studies showed that those with high psychosocial risk factors were more likely to engage in risky life behaviors such as smoking, drinking, physical inactivity, which also contributed to higher risk of stroke [46].The impact of psychosocial factors on stroke risk and its mechanism are worthy of further investigation. One implication of our findings is that future stroke prevention programs may benefit from interventions targeted at reducing psychosocial risk factors, which is both theoretically modifiable and practically feasible.

## Limitation

One major limitation of the current study was the cross-sectional study design that precludes establishment of causal relationship between quality of life and stroke risk. Based on the past evidence showing both directions, it is likely that the association between quality of life and stroke risk are bidirectional. Future longitudinal study with more complex design is needed to further test this hypothesis. Another limitation is that our sample came from urban communities in Shanghai city, which has higher economic development and high health literacy than rural communities and other less-developed cities and provinces of China. Our results may not be representative to other parts of China. It may be worthwhile to conduct national study that include both rural and urban communities from various parts of China to gain a whole picture of stroke risk as well as make comparisons by regions. A third limitation is that we only examined the impact of one psychosocial factor-quality of life on stroke risk, future studies may benefit from adding more other psychosocial factors such as life events, perceived stress, and social support to see their impacts on stroke risk, which are still under-investigated.

## Conclusion

In conclusion, this study represents an attempt to better understand stroke risk and the association between quality of life and stroke risk in an urban sample of Shanghai residents. Our results showed that one third of residents were at middle or high risk for stroke, with the top three risk factors being physical inactivity, hypertension, and smoking. These findings suggest that future stroke prevention program may benefit from interventions targeted at improving those risk factors such as increasing physical activity, early diagnosis and treatment of hypertension, and smoking cessation. Quality of life was significantly independently associated with stroke risk, with lower quality of life associated with high stroke risk. This finding adds support to the bidirectional relationship between stroke risk and an important modifiable psychosocial factor-quality of life, which provides significant implications for development of psychosocial intervention programs for stroke prevention and after care.

## Data Availability

The data is available upon reasonable request.
